# Characterising timing and pattern of relapse following surgery for localised oesophagogastric adenocarcinoma: a retrospective study

**DOI:** 10.1186/s12885-016-2145-0

**Published:** 2016-02-17

**Authors:** Sing Yu Moorcraft, Elisa Fontana, David Cunningham, Clare Peckitt, Tom Waddell, Elizabeth C. Smyth, William Allum, Jeremy Thompson, Sheela Rao, David Watkins, Naureen Starling, Ian Chau

**Affiliations:** The Royal Marsden NHS Foundation Trust, London and Surrey, United Kingdom

**Keywords:** Follow-up, Gastric cancer, Oesophageal cancer, Recurrence, Surveillance

## Abstract

**Background:**

Oesophagogastric adenocarcinoma (OGA) has a poor prognosis, even for patients with operable disease. However, the optimal surveillance strategy following surgery is unknown.

**Methods:**

We performed a retrospective review of all patients with OGA who had undergone surgery with radical intent at the Royal Marsden between January 2001 and December 2010.

**Results:**

Of the 360 patients with OGA who underwent potentially curative surgery, 100/214 patients (47 %) with oesophageal/gastro-oesophageal junction (GOJ) adenocarcinoma and 47/146 patients (32 %) with gastric adenocarcinoma developed recurrent disease. 51, 79 and 92 % of relapses occurred within 1, 2 and 3 years respectively and the majority of patients relapsed at distant sites. Of the patients who relapsed, 67 % (67/100) with oesophageal/GOJ adenocarcinoma and 72 % of patients with gastric cancer (34/47) were symptomatic at the time of relapse. The majority of asymptomatic relapses were first detected by a rise in tumour markers. There was no difference in disease-free survival between asymptomatic and symptomatic patients, but asymptomatic patients were more likely to receive further treatment and had a longer survival beyond relapse.

**Conclusion:**

The majority of relapses occur within the first 3 years and at distant sites. Monitoring of tumour markers should be considered as part of a surveillance program.

## Background

Oesophagogastric adenocarcinoma (OGA) has a poor prognosis, even in patients who present with localised disease. Over time, staging has become more accurate, leading to improvements in the selection of patients for surgery, and treatment has improved, with peri-operative chemotherapy becoming a standard of care in the United Kingdom, based on a 5–year overall survival (OS) of 36 - 38 % compared to 23–24 % for surgery alone [1, 2]. Worldwide, other treatment options include neoadjuvant or adjuvant chemoradiotherapy or chemotherapy. Extended lymph node dissection (D2 lymphadenectomy) has also become a standard of care due to evidence that this leads to a reduced rate of gastric cancer-related deaths [3]. In addition, the treatment of metastatic OGA has improved, with the addition of new treatment options. For example, trastuzumab is used in the first-line treatment of HER2 positive gastric cancer [4], second-line chemotherapy is now a standard of care [5] and benefit has also been seen with the anti-angiogenic agent ramucirumab [6].

In theory, early detection of disease relapse could lead to improved outcomes for patients. However, the optimal follow-up schedule for patients after potentially curative resection for OGA is not yet determined and there are significant variations between guidelines. For example, the National Comprehensive Cancer Network guidelines recommend performing a history and physical examination every 3–6 months for 1–2 years, then every 6–12 months for 3–5 years and then annually, with other investigations being done as clinically indicated [7], whereas other guidelines state that there is no evidence that intensive follow-up impacts on outcomes [8–10]. This leaves clinicians with uncertainty regarding the optimal management of these patients.

We conducted a retrospective analysis to investigate patterns of relapse following resection for OGA to assist in formulating an optimal surveillance strategy for these patients.

## Methods

This project was classified as a service evaluation by our institution’s Committee for Clinical Research as the aim of the project was to evaluate our institution’s follow-up strategy for patients undergoing surgery for OGA. Therefore, in accordance with guidance from the National Health Service (NHS) Health Research Authority, specific patient consent and ethical approval was not required. After approval from our institution’s Committee for Clinical Research (SE3407), we searched the Royal Marsden (RM) electronic medical record system for patients with a diagnosis of oesophageal, gastro-oesophageal junction (GOJ) or gastric adenocarcinoma who had undergone surgery with radical intent between January 2001 and December 2010. Patients who were followed up in another hospital, patients for whom no data was available apart from the date of surgery and patients who were found to have unresectable metastatic disease at the time of surgery were excluded.

**Fig. 1:**
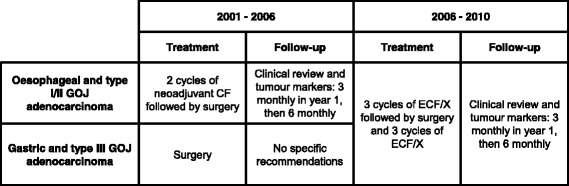
Changes in the treatment and surveillance paradigms for oesophageal, GOJ and gastric adenocarcinomas. CF = cisplatin and 5-fluorouracil, ECF/X = epirubicin, cisplatin and 5-fluorouracil/capecitabine

Prior to 2006, our institution’s policy for patients with oesophageal/type I/II GOJ cancer was 2 cycles of neoadjuvant chemotherapy with cisplatin and 5-fluorouracil. The follow-up schedule involved clinical assessment and tumour markers 3 monthly for the first year, then 6 monthly, with endoscopies or CT scans performed as clinically indicated. Patients with operable type III GOJ/gastric cancer underwent surgery alone, unless they were participating in a clinical trial, and there were no specific follow-up recommendations. From 2006, our institution’s policy changed to 3 cycles of neoadjuvant chemotherapy with epirubicin, cisplatin and 5-fluorouracil/capecitabine (ECF/X) followed by surgery and a further 3 cycles of ECF/X for oesophageal, GOJ and gastric adenocarcinoma. Follow-up continued as per our previous standard practice for oesophageal cancer. The treatment and surveillance paradigms are summarised in Fig. 1. Patients with oesophageal or type I/II GOJ adenocarcinoma underwent oesophagogastrectomy and patients with gastric cancer underwent total or subtotal gastrectomy. Nodal dissection tended to be D2 throughout the study period.

Clinical information, including patient demographics, clinical characteristics, outcomes and details of first relapse (including date, site, symptoms, method of relapse detection, CEA and CA19-9) were retrospectively collected from patient records. Patients were categorised as having local relapse (recurrence at the anastomosis) or distant relapse (recurrence at distant sites or regional lymph nodes). Symptomatic relapse was defined as the presence of patient-reported symptoms triggering further investigations, whereas asymptomatic relapse was defined as relapse detected by a routine radiological, laboratory or endoscopic investigation that was not prompted by any clinical concerns.

### Statistical analysis

Disease-free survival (DFS) was calculated from the date of surgery to the date of death or relapse at any site. OS was calculated from the date of surgery to the date of death. Survival beyond relapse (SBR) was calculated from the date of relapse at any site to the date of death from any cause. Patients who were still alive and event free were censored at the time of last follow-up.

Survival rates were calculated using Kaplan Meier methods. Association of survival outcomes with baseline prognostic factors was determined by Cox regression univariate analysis, with hazard ratios being presented with 95 % confidence intervals. Factors included in the univariate analysis were peri-operative treatment (pre-operative, post-operative or both vs surgery alone), pathological T-stage (T0-2 vs T3/4) and N-stage (N0 vs N1-3), differentiation (well/moderate vs poor), resection margin (R0 vs R1/2, includes both circumferential and longitudinal margins), type of relapse (local vs distant vs both), elevated tumour markers pre-operatively (yes vs no) and symptoms at time of recurrence (yes vs no). Significant variables were included in a multivariate analysis.

## Results

### Patient characteristics

Between January 2001 and December 2010, 360 patients with oesophagogastric adenocarcinoma (214 patients with oesophageal/GOJ tumours and 146 patients with gastric tumours) underwent surgery with curative intent at RM. Baseline demographic, clinical and pathological characteristics are shown in Table 1.

**Table 1:** Baseline characteristics, initial treatment details and pathological characteristics of patients with oesophagogastric adenocarcinoma who underwent surgery with curative intent

	Oesophageal/GOJ (*n* = 214)	Gastric (*n* = 146)
	N (%)	N (%)
Gender		
Male	188 (88 %)	98 (67 %)
Female	26 (12 %)	48 (33 %)
Median age (range)	64 years (33–83)	70 years (24–89)
ECOG performance status		
0	58 (27 %)	40 (27 %)
1	69 (32 %)	41 (28 %)
2	2 (1 %)	10 (7 %)
Unknown	85 (40 %)	55 (38 %)
Site of primary tumour		
Oesophagus	29 (14 %)	-
Type 1 GOJ	77 (36 %)	-
Type 2 GOJ	63 (29 %)	-
Type 3 GOJ	45 (21 %)	-
Gastric	-	146 (100 %)
Elevated tumour markers pre-operatively		
Yes	61 (29 %)	27 (19 %)
No	122 (57 %)	75 (51 %)
Unknown	31 (14 %)	44 (30 %)
Baseline PET performed		
Yes	69 (32 %)	24 (16 %)
Treatment		
Neoadjuvant^a^	125 (58 %)	30 (21 %)
Peri-operative^b^	51 (24 %)	56 (38 %)
Adjuvant	5 (2 %)	7 (5 %)
Surgery only	33 (15 %)	53 (36 %)
Surgery		
Oesophagogastrectomy	178 (83 %)	3 (2 %)
Total gastrectomy	35 (16 %)	51 (35 %)
Sub-total gastrectomy	1 (1 %)	92 (63 %)
Differentiation		
Well	8 (4 %)	4 (3 %)
Moderate	84 (39 %)	43 (30 %)
Poor	107 (50 %)	94 (64 %)
Unknown	15 (7 %)	5 (3 %)
T stage		
T0	11 (5 %)	7 (5 %)
T1	48 (22 %)	34 (23 %)
T2	53 (25 %)	66 (45 %)
T3	89 (42 %)	27 (19 %)
T4	10 (5 %)	9 (6 %)
Tx	3 (1 %)	3 (2 %)
N stage		
N0	105 (49 %)	72 (49 %)
N1	92 (43 %)	40 (27 %)
N2	10 (5 %)	20 (14 %)
N3	3 (1 %)	11 (8 %)
Nx	4 (2 %)	3 (2 %)
M stage^c^		
M0	204 (95 %)	139 (95 %)
M1	5 (2 %)	4 (3 %)
Mx	5 (2 %)	3 (2 %)
Number of lymph nodes resected		
Median (range)	28 (4–76)	24 (3–69)
Number of positive lymph nodes		
Median (range)	1 (0–33)	1 (0–35)
Resection margin		
R0	161 (75 %)	135 (92 %)
R1	47 (22 %)	7 (5 %)
R2	0 (0 %)	0 (0 %)
unknown	6 (3 %)	4 (3 %)

^a^ 2 patients received pre-operative chemotherapy followed by pre-operative chemoradiotherapy, ^b^ 19 patients received pre-operative chemotherapy and post-operative chemoradiotherapy, ^c^ M1 = patients with resected metastatic disease (usually peritoneal)

### Survival outcomes

**Fig. 2:**
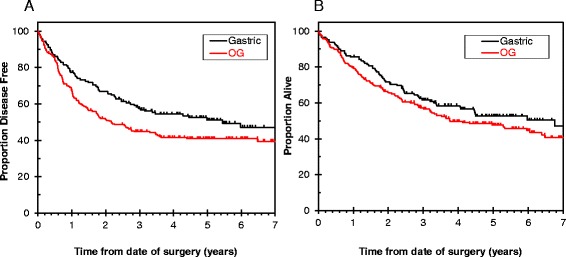
Disease free survival and overall survival for patients who had radical surgery for oesophageal/GOJ (OG) and gastric adenocarcinoma. **a**: Disease –free survival. **b**: Overall survival. (colour figure)

After a median follow-up of 61.7 months, 100 patients (47 %) with oesophageal/GOJ adenocarcinoma and 47 patients (32 %) with gastric adenocarcinoma had developed local and/or distant recurrence. Patients with oesophageal/GOJ adenocarcinoma had a median DFS of 26.1 months (95 % CI 17.7–41.9) and median OS of 45.2 months (95 % CI 36.1–76.7); whereas patients with gastric adenocarcinoma had a median DFS of 65.4 (95 % CI 34.8–99.2) and median OS of 81.2 months (95 % CI 40.6–99.2) (see Fig. 2). The 5-year OS rate was 47.6 % (95 % CI 40.5–54.4) for oesophageal/GOJ adenocarcinoma and 52.6 % (95 % CI 43.7–60.8) for gastric adenocarcinoma. Median SBR was 8.1 months (95 % CI 6.1–13.4) and 5.9 months (95 % CI 3.4–8.2) for oesophageal/GOJ and gastric adenocarcinoma respectively.

### Patterns of relapse

The majority of relapses occurred at distant sites and occurred within the first 3 years following surgery, with 51, 79 and 92 % of relapses occurring within 1, 2 and 3 years respectively (see Table 2). Sixty-three patients (63 %) with oesophageal/GOJ adenocarcinoma and 24 patients (51 %) with gastric cancer had elevated tumour markers at the time of relapse. Of the 11 patients with anastomotic relapse only, 7 received further treatment (chemotherapy: 3 patients, chemotherapy followed by radiotherapy: 2 patients, radiotherapy: 1 patient, chemoradiotherapy and surgery: 1 patient).

**Table 2:** Patterns of disease recurrence and treatment of recurrent disease

	Oesophageal/GOJ (*n* = 100)	Gastric (*n* = 47)
	N (%)	N (%)
Time to relapse		
< 12 months	53 (53 %)	22 (47 %)
12–24 months	29 (29 %)	12 (25 %)
24–36 months	12 (12 %)	7 (15 %)
> 36 months	6 (6 %)	6 (13 %)
Relapse type		
Local	7 (7 %)	4 (9 %)
Distant	79 (79 %)	37 (79 %)
Both	14 (14 %)	6 (13 %)
Site of relapse^a^		
Lymph nodes	52 (52 %)	14 (30 %)
Anastomosis	21 (21 %)	10 (21 %)
Peritoneum	16 (16 %)	18 (38 %)
Liver	18 (18 %)	9 (19 %)
Bone	12 (12 %)	4 (9 %)
Abdominal wall	3 (3 %)	5 (11 %)
Lung	10 (10 %)	2 (4 %)
Brain	10 (10 %)	0 (0 %)
Mediastinum	9 (9 %)	1 (2 %)
Other	8 (8 %)	5 (11 %)
Elevated tumour markers at relapse		
Yes	63 (63 %)	24 (51 %)
No	24 (24 %)	16 (34 %)
Unknown	13 (13 %)	7 (15 %)
Symptoms at time of relapse		
Yes	67 (67 %)	34 (72 %)
How relapse was first detected in asymptomatic patients	(*n* = 33)	(*n* = 12)
Routine tumour markers	22 (67 %)	4 (33 %)
Routine CT	6 (18 %)	4 (33 %)
Concurrent routine CT/ markers	1 (3 %)	3 (25 %)
Endoscopy	2 (6 %)	1 (8 %)
Other	2 (6 %)	0 (0 %)
ECOG performance status at relapse		
0	12 (12 %)	3 (6 %)
1	13 (13 %)	7 (15 %)
2	4 (4 %)	2 (4 %)
3–4	8 (8 %)	4 (9 %)
Unknown	63 (63 %)	31 (66 %)
Further treatment for recurrent disease		
Yes	72 (72 %)	22 (47 %)
Type of treatment for recurrent disease^b^		
Chemotherapy	63 (88 %)	19 (86 %)
Radiotherapy	21 (29 %)	3 (14 %)
Chemoradiotherapy	1 (1 %)	0 (0 %)
Surgery	5 (7 %)	1 (5 %)

^a^ Relapse may have occurred at more than one site

^b^ Patients may have received more than one type of treatment

Sixty-seven patients (67 %) with oesophageal/GOJ adenocarcinoma and 34 patients with gastric cancer (72 %) were symptomatic at the time of relapse. Twenty-six of the asymptomatic patients (58 %) had relapse initially detected via elevated tumour markers. Therefore, elevated tumour markers were the first sign of relapse in 18 % of the 147 patients who relapsed. Occasionally patients had CT scans erroneously arranged as part of routine follow-up and these scans detected relapse in 10 of the asymptomatic patients (22 %) (see Table 2). There were no differences in pathological T or N stage at surgical resection between symptomatic and asymptomatic patients. There was no difference in median DFS between asymptomatic and symptomatic patients with oesophageal/GOJ cancer (*p* = 0.793) or gastric cancer (*p* = 0.259), but asymptomatic patients were more likely to receive further treatment than symptomatic patients (oesophageal/GOJ: 84.5 % vs 65.6 %, *p* = 0.045; gastric: 76.9 % vs 35.3 %, *p* = 0.011) and had a longer SBR (oesophageal/GOJ: 14.6 months vs 5.8 months, HR 1.75, 95 % CI 1.10–2.76, *p* = 0.017; gastric: 10.6 months vs 3.8 months, HR 3.35, 95 % CI 1.55–7.26, *p* = 0.002). Of the 94 patients who received treatment after relapse, SBR was longer in asymptomatic patients compared to symptomatic patients (15.9 months vs 10.7 months, *p* = 0.032).

### Prognostic variables

Univariate analyses (see Table 3), demonstrated that differentiation, pathological T-stage and pathological N-stage were prognostic for DFS and OS for both oesophageal/GOJ and gastric adenocarcinoma and type of relapse was prognostic for OS. In addition, resection margin (R0 vs R1/2) was prognostic for DFS and OS for oesophageal/GOJ adenocarcinoma and there was a trend towards positivity for gastric cancer, although this did not reach statistical significance. The results of a multivariate analysis are shown in Table 4.

**Table 3:** Univariate analysis of disease-free and overall survival

Disease-free survival								
	Oesophageal/GOJ adenocarcinoma				Gastric adenocarcinoma			
Covariate	N	Median DFS (months, 95 % CI)	Hazard ratio (95 % CI)	P -value	N	Median DFS (months, 95 % CI)	Hazard ratio (95 % CI)	P -value
Elevated tumour markers								
No	24	12.2 (8.8–16.2)	1.0	0.794	16	10.8 (5.0–13.7)	1.0	0.081
Yes	63	11.8 (8.4–13.6)	1.07 (0.66–1.72)		24	15.0 (10.6–24.8)	0.56 (0.29–1.08)	
Differentiation								
Poor	107	12.3 (8.8–20.7)	1.0	<0.001	94	37.9 (21.5–71.8)	1.0	0.020
Moderate/well	92	85.9 (33.1 – NA)	0.40 (0.27–0.58)		47	99.2 (36.2 – NA)	0.54 (0.32–0.91)	
Pathological T-stage								
T0-2	112	111.7 (77.7 – NA)	1.0	<0.001	107	86.9 (51.4–99.6)	1.0	0.010
T3/4	97	12.2 (8.7–18.0)	2.89 (2.00–4.18)		36	21.5 (12.7–40.5)	1.91 (1.16–3.12)	
Pathological N-stage								
N0	105	111.7 (77.7 – NA)	1.0	<0.001	72	87.1 (86.9 – NA)	1.0	<0.001
N1-3	105	11.8 (8.4–15.7)	3.38 (2.32–4.94)		71	21.1 (12.7 - 38.0)	3.10 (1.89–5.10)	
Resection margin								
R0	161	77.7 (26.1 – NA)	1.0	<0.001	135	71.8 (35.6–99.6)	1.0	0.080
R1/R2	47	8.7 (7.0–14.8)	2.87 (1.96–4.20)		7	13.2 (0.3 – NA)	2.13 (0.91–4.98)	
Presence of symptoms at time of relapse								
No	33	10.9 (7.9–14.8)	1.0	0.793	13	11.5 (4.8–21.5)	1.0	0.259
Yes	67	11.8 (7.2–12.4)	1.06 (0.70–1.61)		34	13.2 (8.1–20.6)	0.68 (0.35–1.32)	
Neoadjuvant, adjuvant or perioperative therapy								
No	33	140.0 (111.7 – NA)	1.0	0.001	53	34.1 (13.1–87.1)	1.0	0.100
Yes	181	20.9 (14.3–27.2)	3.57 (1.74–7.31)		93	86.9 (41.7 – NA)	0.67 (0.42–1.08)	
Overall survival								
	Oesophageal/GOJ adenocarcinoma				Gastric adenocarcinoma			
Covariate	N	Median OS (months, 95 % CI)	Hazard ratio (95 % CI)	P -value	N	Median OS (months, 95 % CI)	Hazard ratio (95 % CI)	P -value
Elevated tumour markers								
No	24	28.8 (15.2–40.7)	1.0	0.343	16	20.0 (9.6–29.1)	1.0	0.842
Yes	63	22.4 (14.9–31.5)	1.28 (0.77–2.11)		24	22.6 (15.9–34.4)	0.91 (0.49–1.80)	
Differentiation								
Poor	107	21.5 (15.2–33.0)	1.0	<0.001	94	40.5 (28.5–86.9)	1.0	0.011
Moderate/well	92	85.9 (76.7 – NA)	0.37 (0.25–0.55)		47	99.2 (53.7 – NA)	0.50 (0.29–0.85)	
Pathological T-stage								
T0-2	112	111.7 (77.7 – NA)	1.0	<0.001	107	81.2 (53.9–99.6)	1.0	0.002
T3/4	99	27.9 (14.9–35.2)	2.96 (2.00–4.36)		36	29.1 (17.2–40.5)	2.19 (1.33–3.61)	
Pathological N-stage								
N0	105	111.7 (77.7 – NA)	1.0	<0.001	72	87.1 (86.9 – NA)	1.0	<0.001
N1-3	105	25.1 (14.7–34.1)	3.33 (2.23–4.97)		71	28.5 (19.4–48.7)	3.16 (1.90–5.26)	
Resection margin								
R0	161	77.7 (51.9 - NA)	1.0	<0.001	135	81.2 (48.8–99.6)	1.0	0.062
R1/R2	47	13.8 (8.6–36.1)	2.83 (1.91–4.19)		7	17.2 (0.3 - NA)	2.25 (0.96–5.26)	
Type of relapse								
None	114	140.0 (111.7 – NA)	1.0	(<0.001)	99	99.2 (63.9–110.5)	1.0	(<0.001)
Local	5	38.1 (23.8 – NA)	3.72 (1.43–9.67)	0.007	4	20.0 (17.2 - NA)	5.61 (1.93–16.2)	0.001
Distant	79	19.4 (14.4–27.9)	6.66 (4.25–10.4)	<0.001	37	20.9 (15.4–28.5)	7.13 (4.17–12.2)	<0.001
Both	14	26.3 (12.5 – NA)	5.31 (2.55–11.1)	<0.001	6	23.1 (14.0 – NA)	5.88 (2.39–14.5)	<0.001
Presence of symptoms at time of relapse								
No	33	26.3 (22.1–38.9)	1.0	0.071	13	29.1 (15.4–53.7)	1.0	0.137
Yes	66	18.6 (13.2–28.4)	1.52 (0.96–2.41)		34	20.0 (14.7–26.9)	1.68 (0.85–3.36)	
Neoadjuvant, adjuvant or perioperative therapy								
No	33	140 (111.7 – NA)	1.0	0.006	53	34.4 (18.7–87.1)	1.0	0.028
Yes	181	39.8 (28.8–59.6)	2.59 (1.31–5.14)		93	86.9 (53.6 – NA)	0.59 (0.37–0.94)	

*NA* means confidence interval is un-obtainable

**Table 4:** Multivariate analysis of disease-free and overall survival

Disease-free survival	Oesophageal/GOJ		Gastric	
Covariate	Hazard ratio (95 % CI)	*P*-value	Hazard ratio (95%CI)	*P*-value
Differentiation	0.58 (0.39–0.86)	0.007	-	-
N-stage	1.59 (1.05–2.40)	0.028	-	-
T-stage	-	-	1.9 (1.13–3.13)	0.015
Overall survival	Oesophageal/GOJ		Gastric	
Covariate	Hazard ratio (95 % CI)	*P*-value	Hazard ratio (95%CI)	*P*-value
Differentiation	0.47 (0.31–0.72)	0.000	0.45 (0.26–0.78)	0.005
N-stage	1.64 (1.06–2.53)	0.027	-	-
Local relapse	2.92 (1.01–8.48)	0.049	3.98 (1.36–11.69)	0.012
Distant relapse	5.40 (3.28–8.90)	0.000	9.10 (5.13–16.14)	0.000
Local and distant relapse	3.61 (1.61–8.10)	0.002	8.75 (3.44–22.24)	0.000
Neoadjuvant, adjuvant or perioperative therapy	-	-	0.31 (0.19–0.52)	0.000

## Discussion

There are no randomised controlled trials investigating the optimum follow-up strategy for patients undergoing curative resection for OGA and strategies vary significantly. For example, some institutions have intensive surveillance programs involving regular imaging and endoscopy, whereas other institutions have a clinically-based follow-up strategy or no follow-up at all [11–14]. It is important to remember that follow-up is not only about the detection of recurrent disease. Other important aspects of follow-up include helping patients to adjust to the social, physical and psychological consequences of surgery [15], correction of nutritional deficiencies and anaemia [11, 16], providing reassurance to patients and providing a forum for patients to mention any new concerns [11].

In keeping with previously published results, 32 % of patients with gastric adenocarcinoma and 47 % of patients with oesophageal/GOJ adenocarcinoma developed recurrent disease [13, 17–19], with the majority of relapses occurring within the first 3 years. This pattern is similar to other studies, which reported that 46–50 % of relapses occurred within 1 year, 75–80 % within 2 years and 90–94 % within 4 years [13, 14, 18–22]. The greatest benefit from a surveillance program is therefore likely to be in the first few years after surgery, and it may be reasonable to discontinue routine follow-up after this time due to the low risk of recurrence.

The majority of relapses occur at distant sites and only 7 % of relapses occurred at the anastomotic site alone. There are variations in the definition of local relapse as some studies define this as relapse at the anastomosis and others include relapse at local or locoregional lymph nodes. However, previous studies demonstrated that 63–90 % of relapses involve regional or distant sites [1, 14, 17, 18, 20, 21, 23, 24]. This highlights the importance of systemic chemotherapy as this can reduce the risk of metastatic disease and improve OS [1, 2]. Although the univariate analysis did not show an improvement in survival for patients with oesophageal/GOJ adenocarcinoma who received neoadjuvant/perioperative or adjuvant treatment, this may be due to patients with less advanced disease being treated with surgery alone. In keeping with results reported by other patient series, we found that differentiation, lymph node involvement, depth of tumour invasion and resection margin were associated with risk of relapse and OS [13, 17, 19, 21, 23].

Tumour markers can be a useful indicator of relapse. A nationwide Japanese study demonstrated that in gastric cancer, the sensitivity of CEA, CA19-9 and a combination of both for detection of relapse were 66, 55 and 85 % respectively, and the specificity was 81 % for CEA and 94 % for CA19-9 [25]. In a large Korean study, 21 % of relapses detected by regular follow-up were first suspected due to a rise in tumour markers [12], and in our study, the majority of asymptomatic relapses were first detected by routine tumour markers. Tumour markers may rise prior to detection of recurrence by imaging and are particularly useful if elevated at baseline [25, 26]. In the future, newer techniques may become available for the detection of micrometastatic disease. For example, elevated plasma DNA has a higher sensitivity (but lower specificity) than CEA for the detection of recurrent disease [27].

Endoscopy is not part of routine follow-up in our institution. Although endoscopy can be helpful for the detection of surgical complications, such as benign strictures [28] and annual endoscopies following partial gastrectomy have been suggested due to the risk of second malignancies [16], there is no definitive evidence for its role as part of a surveillance strategy. Firstly, as previously discussed, the frequency of local relapse only is low. Secondly, a large study of 1147 patients at Memorial Sloan-Kettering Cancer Centre who underwent regular endoscopies as part of their follow-up schedule showed that only 1 % of asymptomatic recurrences were detected by routine endoscopies and 65 % of patients with peri-anastomotic recurrences were initially suspected by the presence of symptoms [14]. Furthermore, local curative re-resection is usually only possible in a small number of patients [14, 29], and of our 11 patients with anastomotic recurrence, only one subsequently underwent surgery.

Previous studies have shown that although relapse may be detected earlier with intensive surveillance, this does not translate to an OS benefit [20, 30, 31] and earlier diagnosis of recurrent disease could adversely affect patients’ quality of life due to anxiety associated with the knowledge of disease relapse. The management of recurrent disease is a major challenge in OGA. Surgery is not usually appropriate because the majority of patients relapse with metastatic disease, and although small case series have suggested that some patients with small, solitary liver metastases may derive benefit from hepatic resection [32], the overall outcomes remain poor and surgery is unlikely to be curative [20].

In our study, 69 % of patients had symptoms at the time of relapse, which is comparable to that reported by other studies (range 50–78 %) [18, 20, 33–35]. However, in agreement with other studies, there was no significant difference in the median time to recurrence between symptomatic and asymptomatic patients [12, 29, 33–35], and therefore the differences in SBR were not due to lead time bias. It has been suggested that the presence of symptoms at the time of relapse is an adverse prognostic factor, as these patients have a shorter SBR and OS than asymptomatic patients [12, 14, 20, 29, 33–36]. This may indicate that the presence of symptoms is a marker of biological aggressiveness, although results are conflicting as to whether there are any true differences in the sites of recurrence between symptomatic and asymptomatic patients [12, 14, 18, 34–36]. On the other hand, asymptomatic patients were more likely to receive chemotherapy at the time of relapse and this has also been shown in other studies [20, 34, 35], although not in others [36], thereby potentially resulting in improved outcomes. It is uncertain as to the reasons why symptomatic patients were less likely to receive post-recurrence chemotherapy. Although we can postulate that this may be due to these patients having a worse performance status, it was not possible to analyse this due to the number of patients in whom information on performance status was not available, highlighting the limitations of this retrospective study. There may also be other potential confounding variables, patients were not always followed-up exactly in accordance with our unit guidelines and it can be challenging to clearly elucidate the sequence of events from the medical notes.

We suggest that patients are followed up by 3 monthly clinical review for the first year, followed by 6 monthly in years 2 and 3 and then consideration of discharge from follow-up due to the low risk of relapse after 3 years. The role of tumour markers and the benefits of early relapse detection are uncertain, but as CEA and CA19-9 monitoring is relatively inexpensive and straightforward, this could also be performed at the same timepoints. The benefit of this approach could be assessed by a prospective trial that randomised patients to clinical review only versus clinical review plus tumour marker monitoring, although this may be logistically challenging.

## Conclusions

In conclusion, there is currently no proven survival benefit from an intensive surveillance strategy following surgery for OGA. Due to the low frequency of anastomotic relapse alone and the very small proportion of patients with local relapse who are suitable for potentially curative treatment, we feel that a routine endoscopic surveillance program is not currently warranted and we suggest that clinical review is the main component of any surveillance strategy. Monitoring of tumour markers may also be useful for the detection of relapse, however it is unclear whether early detection of relapse is beneficial as curative treatment in this setting is only possible in a very small proportion of patients. Prospective, randomised clinical trials are needed to determine the most effective follow-up strategy.

## Abbreviations

DFS: disease-free survival
ECF/X: Epirubin, cisplatin and fluorouracil/capecitabine
GOJ: gastro-oesophageal junction
OGA: oesophagogastric adenocarcinoma
OS: overall survival
RM: Royal Marsden
SBR: survival beyond relapse
